# The Impact of Overtraining on Anxiety‐Like Behavior and Tight Junction Proteins of the Blood–Brain Barrier in Mice

**DOI:** 10.1002/brb3.71709

**Published:** 2026-08-30

**Authors:** Eunseon Hwang, Minyoung Shin, Taeeon Park, Jeremy J. Kim, Dongheon Yi, Jeayeon Park, Hyo Youl Moon

**Affiliations:** ^1^ Department of Physical Education Seoul National University Seoul South Korea; ^2^ Learning Sciences Research Institute Seoul National University Seoul South Korea; ^3^ Institute of Aging Seoul National University Seoul South Korea

**Keywords:** anxiety‐like behavior, blood–brain barrier, neuroinflammation, Overtraining Syndrome

## Abstract

**Purpose**: Overtraining Syndrome (OTS) is a chronic maladaptive condition characterized by persistent performance decline due to excessive training and inadequate recovery. OTS is associated with mood disturbances, but the underlying neurobiological mechanisms remain unclear. This study investigated whether a 6‐week overtraining (OT) protocol induces anxiety‐like behavior and changes in hippocampal BBB‐related tight junction proteins in mice.

**Methods**: Eight‐week‐old male C57BL/6J mice were assigned to sedentary (SED, *n* = 5), moderate exercise (ME, *n* = 5), or overtraining (OT; downhill treadmill running, initially *n* = 10) groups and completed a 6‐week training protocol. The final OT sample size was *n* = 4 after 6 of the 10 mice were excluded due to tail or hindlimb injuries. Endurance capacity was assessed using an incremental loading test (ILT). Anxiety‐like behaviors were evaluated using the nest building test (NBT), open‐field test (OFT), and elevated plus maze (EPM). Hippocampal tight junction (TJ) proteins zonula occludens‐1 (ZO‐1), occludin, claudin‐5, as well as tumor necrosis factor‐alpha (TNF‐α) were quantified by Western blot analysis.

**Results**: After 6 weeks, endurance performance in the OT group significantly decreased from baseline (*p* < 0.01) and was lower than that of the ME group at Week 6 (*p* < 0.0001). In the quadriceps, TNF‐α protein levels were significantly elevated in the OT group compared with both the SED (*p* < 0.05) and ME groups (*p* < 0.01). In the OFT, OT mice exhibited fewer center entries and reduced time spent in the center zone versus the SED and ME groups (*p* < 0.05), indicating increased anxiety related behavioral change. In the hippocampus, zonula occludens‐1 (ZO‐1) levels were reduced and TNF‐α levels were elevated in OT compared with SED (*p* < 0.01 and *p* < 0.05, respectively), whereas claudin‐5 levels were higher in ME than SED (*p* < 0.05).

**Conclusion**: OT was associated with anxiety‐like behavioral alterations and changes in hippocampal BBB‐related tight junction proteins. Reduced expression of TJ proteins and increased neuroinflammation may contribute to affective symptoms observed in OTS. However, given the small final sample size in the OT group, these findings should be interpreted with caution.

## Graphical abstract

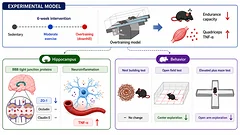

## 1 Introduction

Physical activity is widely recognized as a potent non‐pharmacological intervention for enhancing metabolic health and cognitive function (Cooper et al. 2018). Regular moderate‐intensity exercise has been shown to promote neuroplasticity, reduce systemic inflammation, and improve mood states (Ma 2008; Moon et al. 2012; Vecchio et al. 2018a). However, the relationship between exercise volume and physiological adaptation follows a bell‐shaped curve, whereby training loads that exceed the body's capacity to recover lead to maladaptive responses (Radak et al. 2017). This state, known as Overtraining Syndrome (OTS), is frequently observed in elite athletes and military personnel who undergo high‐intensity training regimens with insufficient recovery (Petibois et al. 2003; Vrijkotte et al. 2019).

In both clinical and experimental settings, OTS is distinguished from functional overreaching by the persistence of performance decrements despite sufficient recovery. In rodent models, prolonged downhill treadmill running has been widely used to induce muscle damage, systemic inflammation, and performance decline, thereby mimicking key features of OTS (Yi et al. 2025).

Clinically, OTS is characterized not only by a stagnation or decrement in physical performance but also by severe mood disturbances, including depression and anxiety, which often precede physiological symptoms (O'Connor et al. 1991; Jahangiri et al. 2019; Armstrong et al. 2022). While the peripheral mechanisms of OTS, such as glycogen depletion and muscle damage, are well‐documented (Costill et al. 1988; Cheng et al. 2020), the neurobiological mechanisms linking excessive physical stress to anxiety‐like behaviors remain poorly understood.

A prevailing theory explaining the systemic effects of OTS is the “cytokine hypothesis,” which posits that repetitive tissue trauma induces a chronic systemic inflammatory response involving elevated levels of pro‐inflammatory cytokines such as tumor necrosis factor‐alpha (TNF‐α) and interleukin‐6 (IL‐6) (Smith 2000; Robson 2003; Kreher and Schwartz 2012). Under physiological conditions, the brain is shielded from peripheral inflammation by the blood–brain barrier (BBB), a selective permeability barrier formed by brain microvascular endothelial cells connected by tight junction (TJ) proteins, including Zonula Occludens‐1 (ZO‐1), Occludin, and Claudin‐5 (Daneman and Prat 2015). However, sustained systemic inflammation can compromise BBB integrity, allowing cytokines and immune cells to infiltrate the brain parenchyma (Takata et al. 2021; Galea 2021; Wu et al. 2022). Disruption of the neurovascular unit is increasingly implicated in the pathophysiology of psychiatric disorders (Welcome 2020; Greene et al. 2020; Sun et al. 2024). Recent studies suggest that stress‐induced BBB breakdown in the hippocampus, a region critical for emotional regulation, promotes neuroinflammation and the development of anxiety‐like behaviors (de Paula et al. 2021; Wu et al. 2023; Wu and Zhang 2023). The hippocampus was selected as a primary region of interest due to its high metabolic demand, sensitivity to inflammatory cytokines, and established role in stress‐related emotional regulation and hypothalamic–pituitary–adrenal (HPA) axis feedback control (Mah et al. 2016; Wu and Zhang 2023). Despite the established link between psychological stress and BBB dysfunction, few studies have examined whether the physiological stress of overtraining (OT) exerts similar deleterious effects on the BBB. It remains unclear how excessive training loads combined with insufficient recovery alter BBB integrity and whether these changes are associated with anxiety in OTS. While moderate exercise is known to strengthen BBB integrity through antioxidant and anti‐inflammatory pathways (Małkiewicz et al. 2019; CHANG et al. 2025), we hypothesize that OT disrupts this barrier via cytokine‐mediated degradation of TJ proteins.

Therefore, the present study aimed to investigate the effects of a 6‐week OT protocol on anxiety‐like behaviors, hippocampal neuroinflammation, and BBB integrity in mice. Specifically, we assessed the protein levels of ZO‐1, Occludin, and Claudin‐5 in the hippocampus and examined their relationship with TNF‐α levels and behavioral outcomes. By contrasting these effects with those of a moderate exercise regimen, this study seeks to elucidate the neurobiological underpinnings of OT‐induced anxiety and provide preliminary evidence of BBB‐related molecular alterations in association with OT as a potential pathological mechanism in OTS.

## 2 Methods

### 2.1 Animal Experiment

Eight‐week‐old male C57BL/6J mice were housed under controlled conditions at 22°C–25°C with 40%–60% humidity and a 12‐h light/dark cycle and were provided ad libitum access to food and water. Mice were randomly divided into three groups: Sedentary (SED, *n =* 5), Moderate exercise (ME, *n =* 5), and Overtraining (OT, *n =* 10). During the protocol, six mice in the OT group developed tail or hindlimb injuries and were excluded from the analysis to avoid confounding effects on physical performance, resulting in a final sample size of four mice in the OT group. Each mouse was housed individually and weighed weekly. All procedures were approved by the Institutional Animal Care and Use Committee of Seoul National University (SNU‐240510‐4‐2).

### 2.2 Experimental Design

**FIGURE 1:**
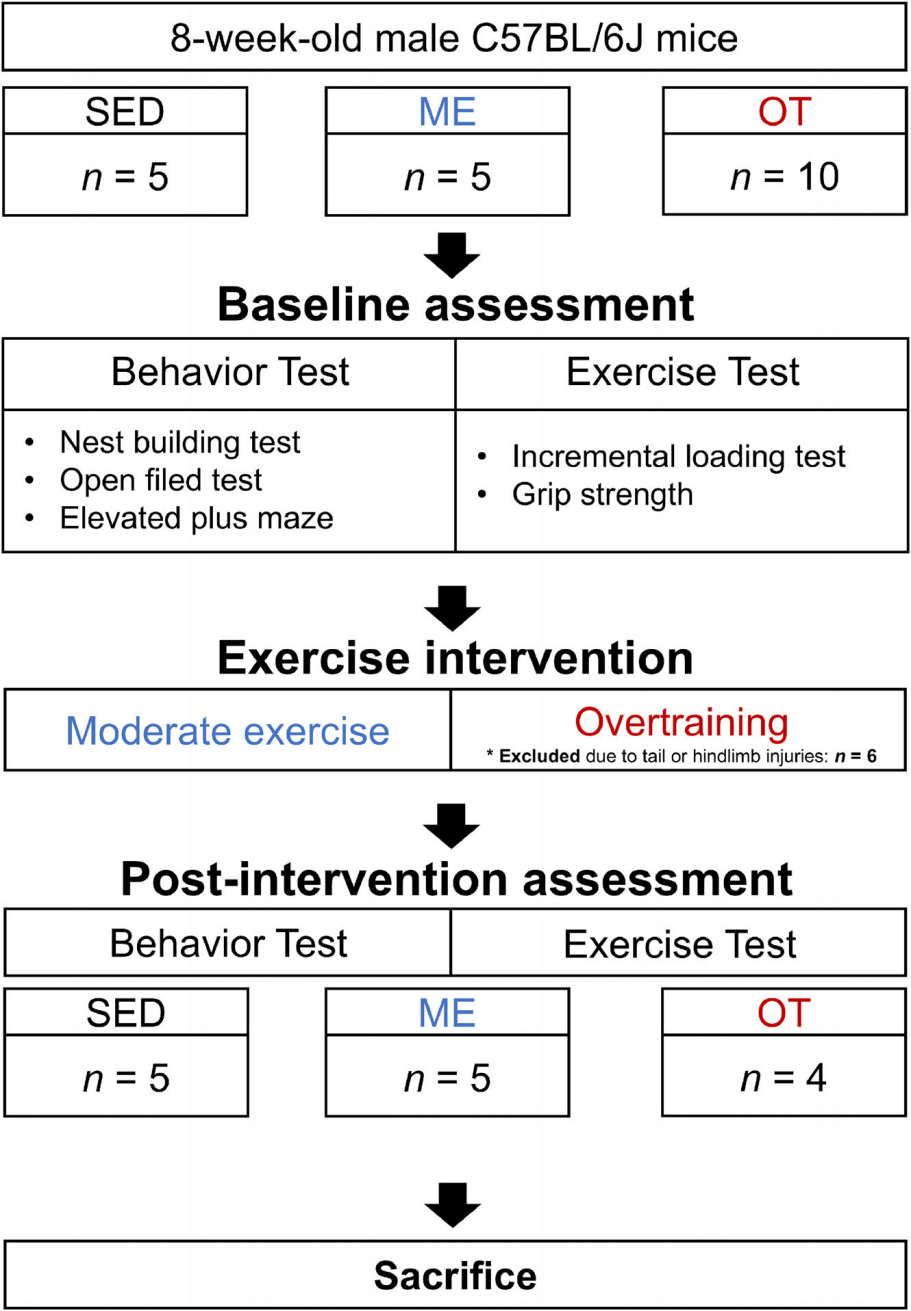
Experimental flow chart of the study.

An overview of the experimental design is shown in Figure 1.

### 2.3 Exercise Protocol

#### 2.3.1 Moderate Exercise Protocol

The moderate exercise program lasted 6 weeks and was adapted from the protocol of Pereira (2015). The exercise protocol is summarized in Table 1.

**TABLE 1:** Moderate exercise protocol.

Moderate exercise					
Week	Intensity (%EV)	Duration (min)	Session/day	Recovery between sessions (h)	Treadmill grade (down, %)
0–6	60%	50	1	24	0

#### 2.3.2 Overtraining Protocol

The OT protocol was adapted from Ferreira (2015) and performed for 6 weeks. During Week 1, mice completed one training session per day for 6 consecutive days. The first 3 training days were performed on a level treadmill (0% grade), followed by 3 days of downhill running (14% grade). Beginning in Week 2, training load was progressively increased through higher running intensity, extended duration, additional daily sessions, and the use of downhill running. The detailed protocol is presented in Table 2.

**TABLE 2:** Overtraining protocol.

Overtraining						
Week	Training period	Intensity (%EV)	Duration (min)	Session/day	Recovery between sessions (h)	Treadmill grade (down, %)
1	Days 1–3	60%	60	1	24	0
	Days 4–6	60%	60	1	24	14
2	Days 1–6	60%	60	2	4	14
3	Days 1–6	70%	60	2	4	14
4	Days 1–6	70%	60	2	4	14
5	Days 1–6	75%	75	2	4	14
6	Days 1–6	75%	75	2	4	14

### 2.4 Exercise Performance Evaluation

#### 2.4.1 Incremental Loading Test (ILT)

A modified ILT, adapted from Ferreira (2015), was conducted 48 h before the initiation of training (Week 0) to establish exercise intensity and 24 h after the final training session of Week 6 to evaluate performance. The test began at 6 m/min with no incline, and speed was increased by 1 m/min every minute. Exhaustion was defined as contacting the treadmill's end 5 times within 1 min. The exhaustion velocity (m/min) was used both to determine training intensities for the moderate exercise and OT protocols and to assess performance capacity.

#### 2.4.2 4‐Paw Grip Strength

Muscle strength was evaluated using a grip strength meter (Bioseb, Vitrolles, France) that measured 4‐paw grip force. Mice were gently pulled by the tail in a horizontal direction until the grip was released, and the peak force (g) was recorded. Each mouse performed three trials with 5‐min intervals, and the mean value was calculated. Data were normalized to body weight.

### 2.5 Behavioral Assessments

#### 2.5.1 Nest Building Test (NBT)

The NBT was conducted once a week, 24 h after the final training session. Each cage received 3 g pressed cotton squares (SOOSUNG, Yangsan, Republic of Korea) as nesting material. Nest quality was assessed between 09:00 and 18:00 using the Deacon method, and scores were averaged from 3 independent evaluators blinded to the experimental conditions.

#### 2.5.2 Open‐Field Test (OFT)

The OFT was performed in an 80 × 80 × 30 cm apparatus divided into four equal compartments (40 × 40 cm each) and fitted with a video‐tracking camera. Each mice was placed at the center and monitored for 10 min. The arena was cleaned with 70% ethanol after each trial. Behavioral data were analyzed using ANY‐maze software (Stoelting Co., Wood Dale, USA). The center zone was defined as the central 20 × 20 cm area of each compartment, and the remaining area was defined as the peripheral zone.

#### 2.5.3 Elevated Plus Maze (EPM)

After all mice had completed the OFT, the EPM was conducted. The apparatus consisted of two open arms (30 × 5 cm), two closed arms (30 × 5 cm) enclosed by 15‐cm‐high walls, and a central platform (5 × 5 cm), elevated 55 cm above the floor.

Each mouse was placed in the central area of the maze, and behavior was recorded for 5 min with an overhead camera. After each trial, the maze was cleaned with 70% ethanol. Behavioral data were analyzed using ANY‐maze software (Stoelting Co., Wood Dale, USA). An arm entry was defined as the animal's center entering an arm. Time spent and entries in the open and closed arms, along with total distance traveled, were quantified.

### 2.6 Tissue Collection

At 48 h after the final training session, mice were anesthetized with isoflurane, and blood was collected by cardiac puncture. Following perfusion with 1× phosphate‐buffered saline (PBS), whole brains were removed and hemisected. One hemisphere was stored at −80°C. Skeletal muscles from both hindlimbs were also harvested and stored at −80°C. For western blotting, the hippocampus was rapidly dissected from the brain, and the tibialis anterior (TA) and quadriceps (QUAD) muscles were collected from both hindlimbs. All tissues were homogenized in ice‐cold RIPA lysis buffer (Biosesang, RC2002‐050‐00, Daejeon, Republic of Korea) containing a protease inhibitor (Roche, 04 693 116 001, Basel, Switzerland) and a phosphatase inhibitor (Roche, 04 906 845 001, Basel, Switzerland) for protein extraction. Protein concentrations were determined using the Bradford assay (Bio‐Rad, #500‐0006, California, USA).

### 2.7 Western Blot

Equal amounts of protein were diluted with 5× SDS‐PAGE loading buffer (Biosesang, SF2002‐110‐00, Seoul, Republic of Korea) and distilled water to the same concentration. The samples were then boiled at 95°C for 5 min and cooled on a cold block. Samples (20–35 µg) were loaded onto 8%–12% SDS‐PAGE gels, electrophoresed, and then transferred to PVDF membranes (Merck Millipore, IPVH00010, Darmstadt, Germany). Membranes were blocked with 3% skim milk for 1 h at room temperature (RT) and incubated overnight at 4°C with primary antibodies against Claudin‐5 (1:1000, 34–1600, Invitrogen), ZO‐1 (1:1000, 21773‐1‐AP, Proteintech), Occludin (1:1000, E6B4R, Cell Signaling Technology), TNF‐α (1:1000, 17590‐1‐AP, Proteintech), GAPDH (1:1000, 14C10, Cell Signaling Technology), and β‐actin (1:4200, A5441, Sigma‐Aldrich). The membranes were washed 3 times with TBST (10 min each) and incubated with horseradish peroxidase‐conjugated anti‐rabbit (1:5000, 7074S, Cell Signaling Technology) or anti‐mouse IgG (1:10000, sc‐516102, Santa Cruz Biotechnology) for 1 h at RT. Protein bands were detected using enhanced chemiluminescence (ECL) reagents (Merck Millipore, WBKLS0500, Seoul, Republic of Korea) and quantified with ImageJ software. Target protein expression was normalized to GAPDH or β‐actin.

### 2.8 Statistical Analysis

Statistical analyses were performed using GraphPad Prism version 10.1.2 (GraphPad Software Inc., CA, USA). Group differences in grip strength, muscle weight, quadriceps inflammation, OFT, EPM, NBT, TJ protein expression, and hippocampal neuroinflammation at Week 6 were assessed by one‐way ANOVA. Longitudinal variables, including body weight and ILT performance, were analyzed using two‐way repeated‐measures ANOVA to assess the effects of group, time, and their interaction. Homogeneity of variance for one‐way ANOVA was evaluated using the Brown–Forsythe test. When significant main effects were detected, Bonferroni post hoc tests were conducted for multiple comparisons. Effect sizes were calculated and reported as *η* ^2^ for one‐way ANOVA and partial *η* ^2^ for two‐way repeated‐measures ANOVA (Table S1). A *p*‐value < 0.05 was considered statistically significant. All values are expressed as mean ± standard error of the mean (SEM).

## 3 Results

### 3.1 Effects of Overtraining on Body Weight

**FIGURE 2:**
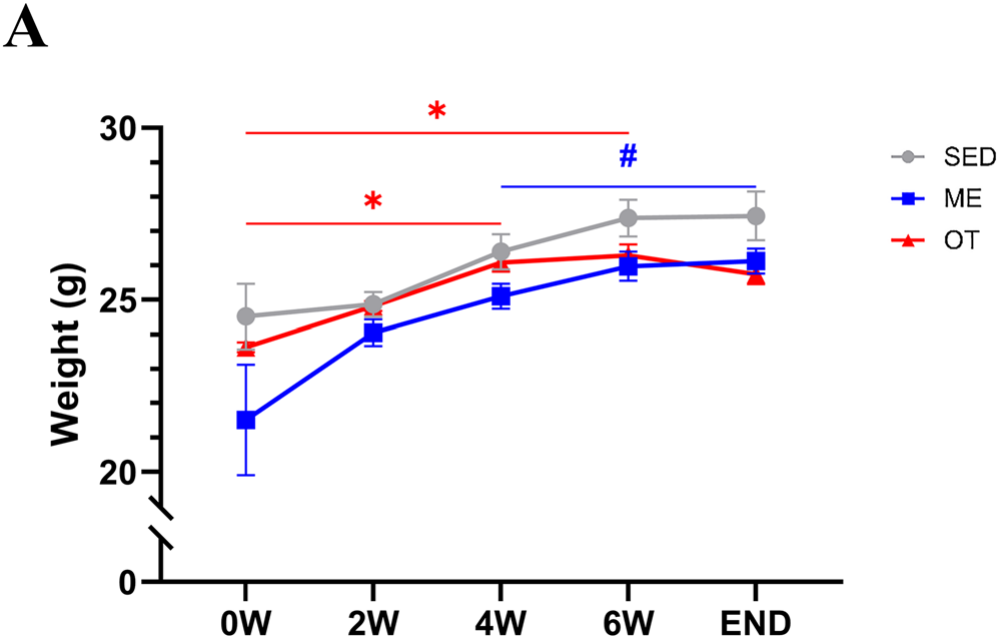
Effects of overtraining on body weight. Body weight measured weekly during the 6‐week intervention period in sedentary (SED, *n =* 5), moderate exercise (ME, *n =* 5), and overtraining (OT, *n =* 4) groups. Data are presented as mean ± SEM. Statistical significance was assessed using two‐way ANOVA with Bonferroni post hoc tests. *p < 0.05 vs. 0 W within the OT group; #*p* < 0.05 vs. 4 W within the ME group. W, week.

Body weight was measured weekly for 6 weeks in the SED, ME, and OT groups (Figure 2). In the OT group, body weight was significantly higher at Week 4 (26.08 ± 0.27 g vs. 23.62 ± 0.14 g; *p* < 0.05) and Week 6 (26.28 ± 0.32 g vs. 23.62 ± 0.14 g; *p* < 0.05) than baseline. The ME group showed a significant increase at END compared to Week 4 (26.12 ± 0.36 vs. 25.1 ± 0.36 g; *p* < 0.05). No significant differences among groups were observed at any time point (*p* > 0.05).

### 3.2 Effect of Overtraining on Physical Performance, Muscle Mass, and Inflammatory Response

**FIGURE 3:**
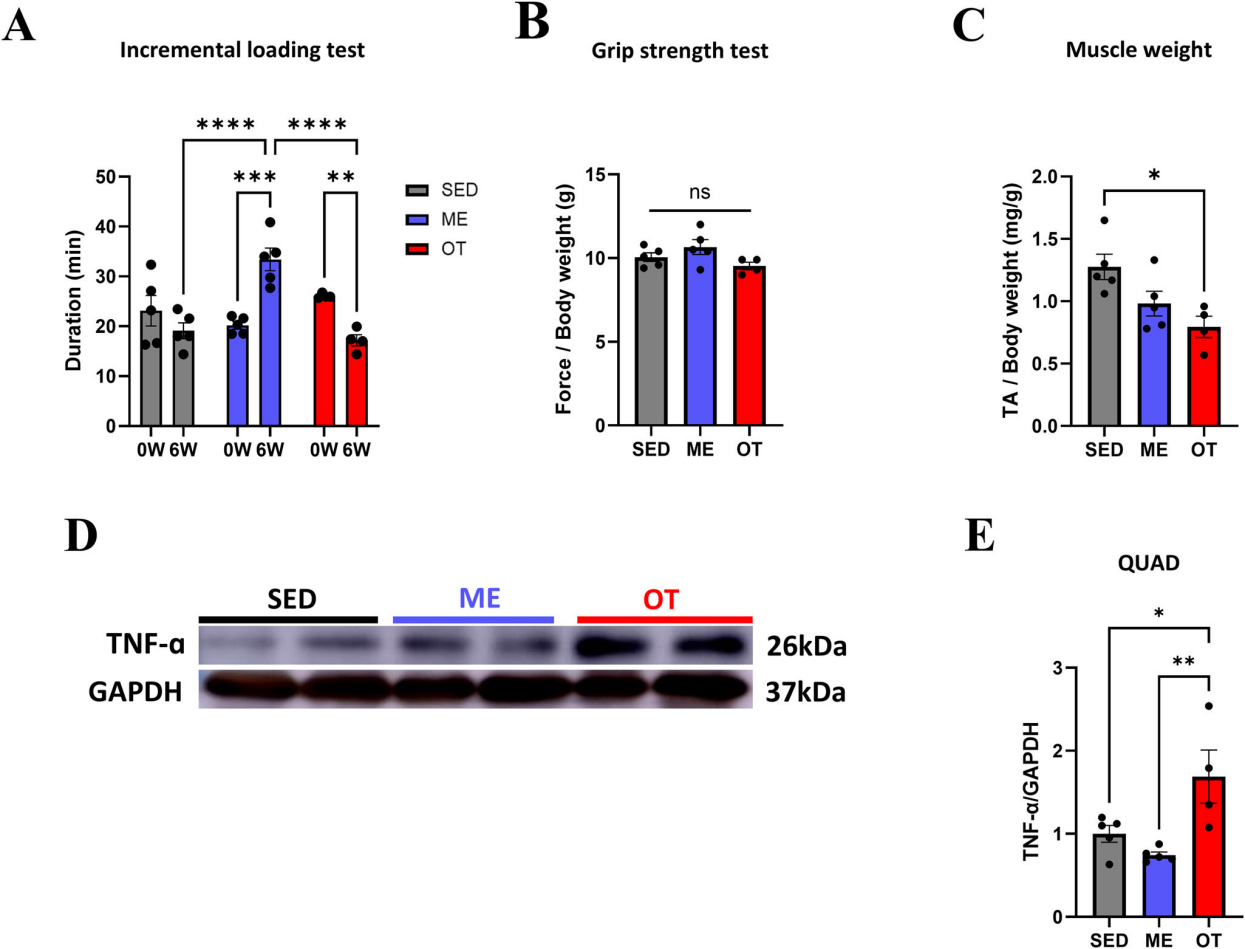
Effects of overtraining on physical performance, muscle mass, and inflammatory response. (A) Duration of the incremental loading test (ILT) in sedentary (SED, *n =* 5), moderate exercise (ME, *n =* 5), and overtraining (OT, *n =* 4) groups at 0 and 6 weeks (W). (B) 4‐paw grip strength and (C) tibialis anterior (TA) muscle weight, both normalized to body weight at 6 W. (D) Representative western blot images and (E) quantified protein levels of TNF‐α in the quadriceps (QUAD), with GAPDH used as a loading control. Statistical significance was assessed using two‐way ANOVA for (A) and one‐way ANOVA for (B–E), followed by Bonferroni post hoc tests (*p < 0.05, **p < 0.01, ****p < 0.0001; ns, not significant). Data are presented as mean ± SEM.

Endurance capacity, measured by ILT, showed no significant differences among groups at Week 0 (*p* > 0.05; Figure 3A). At Week 6, the ME group increased from 20.20 ± 0.76 min at Week 0 to 33.40 ± 2.28 min (*p* < 0.0001), while the OT group decreased from 26.09 ± 0.24 min at Week 0 to 17.19 ± 1.14 min (*p =* 0.01). However, the SED group showed no significant difference between Week 0 and Week 6 (23.13 ± 3.08 min vs. 19.09 ± 1.58 min; *p* > 0.05). By Week 6, endurance performance in the ME group was significantly greater than both the SED (33.40 ± 2.28 min vs. 19.09 ± 1.58 min; *p* < 0.0001) and OT groups (33.40 ± 2.28 min vs. 17.19 ± 1.14 min; *p* < 0.0001). The OT group performed significantly lower than the ME group (17.19 ± 1.14 vs. 33.40 ± 2.28 min; *p* < 0.0001) but did not differ from the SED group (*p* > 0.999). Grip strength at Week 6 showed no significant differences among groups (*p* > 0.05; Figure 3B).

The TA muscle weight, normalized to body weight, was significantly lower in the OT group than in the SED group (0.8 ± 0.09 mg/g vs. 1.28 ± 0.1 mg/g; *p* < 0.05; Figure 3C). No significant difference was observed between the SED and ME groups (*p* > 0.05).

An increased inflammatory response is a representative symptom of OTS. As an indicator of peripheral inflammation, TNF‐α levels in the QUAD were significantly higher in the OT group (1.69 ± 0.32; Figure 3E) compared with the SED (1.00 ± 0.10; *p* < 0.05) and ME groups (0.74 ± 0.08; *p* < 0.01).

### 3.3 Effects of Overtraining on Anxiety‐Like Behaviors

**FIGURE 4:**
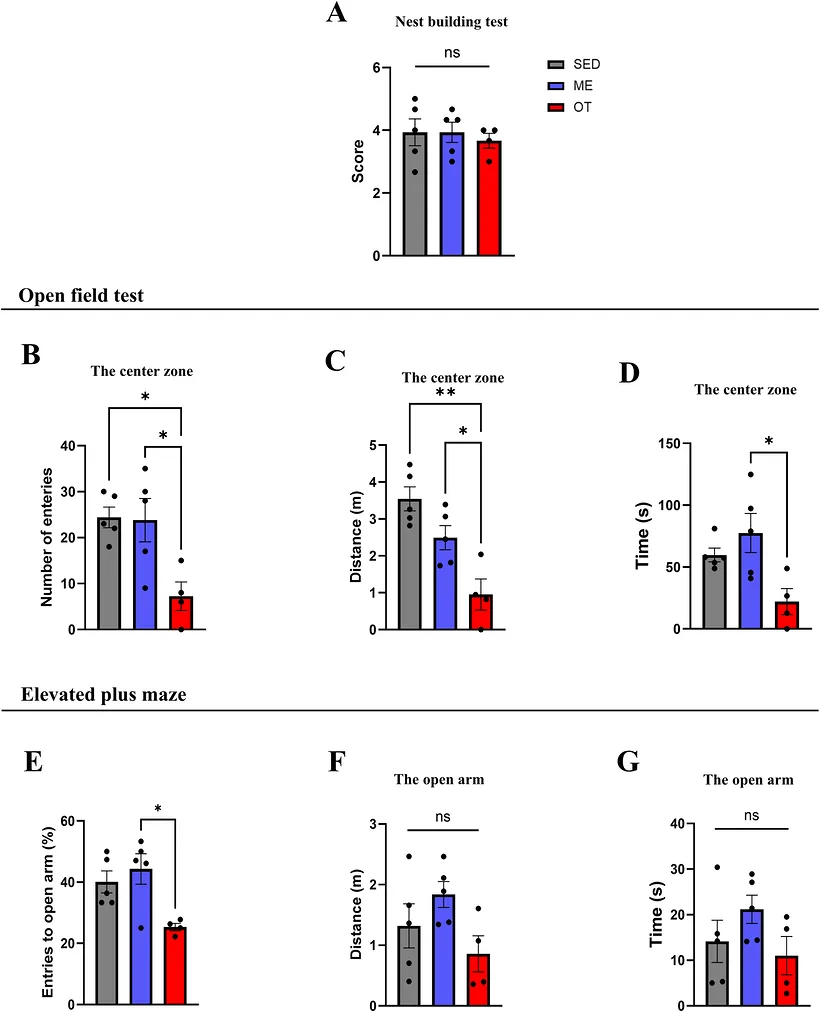
Effects of overtraining on anxiety‐like behaviors. (A) Nest building test (NBT) scores at Week 6 (W). (B–D) Open field test (OFT) results. (E–G) Elevated plus maze (EPM) results: (E) percentage of open arm entries, (F) distance traveled in open arms, and (G) time spent in open arms. Behavioral tests were conducted in sedentary (SED, *n* = 5), moderate exercise (ME, *n* = 5), and overtraining (OT, *n* = 4) groups. Statistical significance was assessed using one‐way ANOVA with Bonferroni post hoc tests (*p < 0.05, **p < 0.01; ns, not significant). Data are presented as mean ± SEM.

To compare the effects of OT on mood states such as anxiety, we assessed mood‐related behaviors using NBT, OFT, and EPM. At Week 6, NBT scores showed no significant differences among the groups (*p* > 0.999; Figure 4A). However, in the OFT, the OT group (7.25 ± 3.09) showed significantly fewer entries into the center zone compared with the SED (24.4 ± 2.25; *p* < 0.05) and ME groups (23.8 ± 4.73; *p* < 0.05; Figure 4B). Similarly, the total distance traveled in the center zone was significantly lower in the OT group (0.95 ± 0.42 m) compared with both the SED (3.55 ± 0.32 m; *p* < 0.01) and ME groups (2.49 ± 0.33 m; *p* < 0.05; Figure 4C). In addition, time spent in the center zone was significantly reduced in the OT group (22.05 ± 10.45 s) compared with the ME group (77.46 ± 15.81 s; *p* < 0.05; Figure 4D). Total distance traveled in the OFT showed a trend toward reduction in the OT group, although the difference did not reach statistical significance (*p* = 0.065).

In the EPM, the ME group showed a significantly higher percentage of open arm entries than the OT group at Week 6 (44 ± 5% vs. 25 ± 1%; *p* < 0.05; Figure 4E). In contrast, no significant group differences were observed in the distance traveled or time spent in the open arms (*p* > 0.05; Figure 4F and G, respectively). Total distance traveled did not differ significantly among the groups.

### 3.4 Effects of Overtraining on Hippocampal Tight Junction Proteins

**FIGURE 5:**
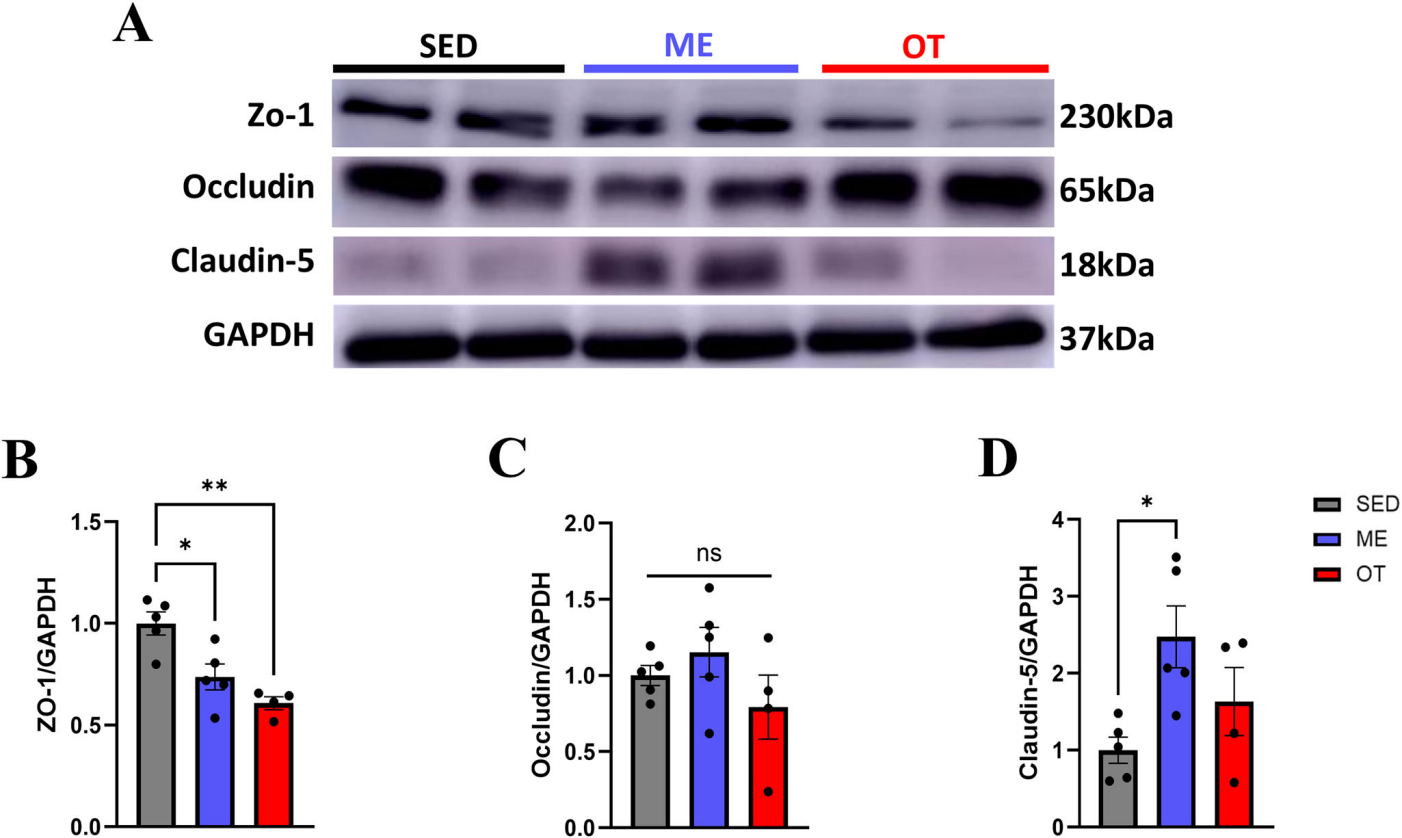
Effects of overtraining on tight junction (TJ) protein expression in the hippocampus. (A) Representative western blot images and (B–D) quantified protein levels of TJ proteins, including (B) ZO‐1, (C) occludin, and (D) claudin‐5, in the hippocampus. GAPDH was used as a loading control. Protein analyses were performed in sedentary (SED, *n =* 5), moderate exercise (ME, *n =* 5), and overtraining (OT, *n =* 4) groups. Statistical significance was assessed using one‐way ANOVA with Bonferroni post hoc tests (*p < 0.05, **p < 0.01; ns, not significant). Data are presented as mean ± SEM.

Hippocampal TJ protein levels were assessed to examine the effects of OT on BBB‐related molecular markers. Western blot analysis showed that ZO‐1 levels were significantly reduced in the OT group (0.61 ± 0.03) compared with the SED group (1.00 ± 0.06; *p* < 0.01; Figure 5B). The ME group also exhibited lower ZO‐1 levels (0.74 ± 0.06) than the SED group (1.00 ± 0.06; *p* < 0.05). In contrast, Occludin levels did not differ significantly among groups (*p* > 0.05; Figure 5C). Claudin‐5 levels were significantly higher in the ME group (2.47 ± 0.40) than in the SED group (1.00 ± 0.17; *p* < 0.05; Figure 5D).

### 3.5 Effects of Overtraining on Hippocampal TNF‐α Expression

**FIGURE 6:**
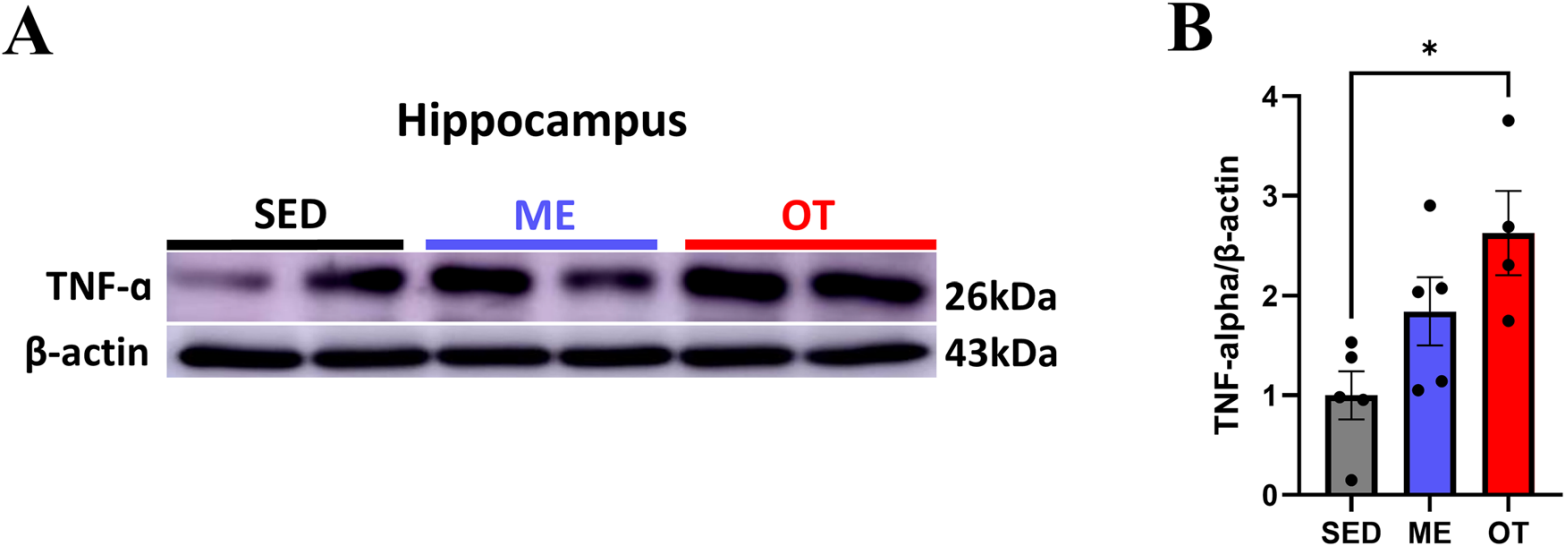
Effect of overtraining on neuroinflammation in the hippocampus. (A) Representative western blot images and (B) quantified protein levels of TNF‐α in the hippocampus. β‐actin was used as a loading control. Protein analyses were performed in sedentary (SED, *n =* 5), moderate exercise (ME, *n =* 5), and overtraining (OT, *n =* 4) groups. Statistical significance was assessed using one‐way ANOVA with Bonferroni post hoc tests (*p < 0.05). Data are presented as mean ± SEM.

To evaluate the effect of OT on neuroinflammation, hippocampal TNF‐α levels were measured by western blot. The OT group showed a significant increase in TNF‐α levels (2.62 ± 0.42) compared with the SED group (1.00 ± 0.24; *p* < 0.05; Figure 6B). No significant difference was observed between the ME (1.84 ± 0.34) and SED groups (*p* > 0.05).

## 4 Discussion

The primary objective of the present study was to examine anxiety‐related behavioral alterations and hippocampal molecular changes associated with OT, with a focus on BBB‐related tight junction proteins and neuroinflammation. Our findings demonstrate that excessive downhill running for 6 weeks not only elicited physiological hallmarks of OTS, including performance decrements and a reduction in relative TA muscle mass, but also led to anxiety‐like behavioral changes, altered the levels of hippocampal BBB‐related proteins, and elevated neuroinflammation.

Specifically, the hippocampus of overtrained mice exhibited a selective decrease in the protein levels of the intracellular scaffolding protein ZO‐1 alongside an increase in TNF‐α protein levels. In contrast, moderate‐intensity exercise induced protective adaptations characterized by increased claudin‐5 protein levels. These findings raise the possibility that the mood disturbances frequently observed in overtrained athletes and military personnel might, at least in part, be related to a neuroimmune crosstalk, in which systemic inflammation may compromise the BBB and increase brain exposure to inflammatory mediators (Müller and Di Benedetto 2025). Furthermore, the divergent protein expression patterns of key BBB proteins, ZO‐1 and claudin‐5, in response to exercise intensity provide significant insights into the fields of exercise physiology and neuroscience.

The physiological and behavioral phenotypes observed in the OT group are consistent with the cytokine hypothesis of OT proposed by Smith (2000). This hypothesis posits that repeated tissue damage in the absence of adequate recovery induces a state of chronic systemic inflammation (Smith 2000). While moderate‐intensity exercise is generally known to promote an anti‐inflammatory milieu and support neuroplasticity (Gleeson et al. 2011; Vecchio et al. 2018b), the excessive eccentric loading paradigm employed in the present study likely shifted this balance toward a chronic pro‐inflammatory state (Newham et al. 1983; Pereira et al. 2015; Vernillo et al. 2017). Notably, the behavioral changes observed in the OT group are consistent with clinical observations reporting an increased prevalence of mood disturbances in overtrained individuals, including athletes (Rice et al. 2019; Reardon et al. 2024). This behavioral alteration may be closely related to the elevated TNF‐α protein levels observed in the hippocampus (Brietzke and Kapczinski 2008; Bauer and Teixeira 2021). Furthermore, the observation that TNF‐α antagonists (e.g., etanercept) can alleviate anxiety‐like behaviors supports the hypothesis that this pro‐inflammatory cytokine may be involved in the pathophysiology of anxiety (Abbott et al. 2015; Alshammari et al. 2020). Although the behavioral changes were not uniform across the OFT, EPM, and NBT, the results suggest that OT influenced several anxiety‐related behavioral measures. Differences in the behavioral domains and sensitivities assessed by each test may partly explain this variability (Pentkowski et al. 2021). In particular, as the NBT reflects broader behavioral alterations rather than anxiety specifically, its findings should be interpreted with caution. In addition, cumulative fatigue, reduced locomotor drive, or other physical burdens inherent to the OT protocol may also have contributed to the behavioral outcomes and should be considered when interpreting these findings.

Recently, proteomic studies using extracellular vesicles derived from the central nervous system suggest that OT is associated with stress‐ and inflammation‐related molecular changes in the brain (Yi et al. 2025). Previous studies have suggested that these alterations may contribute to dysregulation of the hypothalamic–pituitary–adrenal (HPA) axis, as chronic stress and persistent inflammation induced by OT are known to interact with the HPA axis, leading to sustained corticosteroid secretion (Noushad et al. 2021; Armstrong et al. 2022; Nunez et al. 2025). Prolonged exposure to elevated corticosterone levels has been suggested to promote immune cell activation and disrupt BBB integrity, potentially contributing to a cycle of neuroendocrine and neuroinflammatory dysfunction (Welcome 2020; Varanoske et al. 2022; Sharan and Vellapandian 2024).

The hippocampal elevation of TNF‐α is of particular significance, given that this cytokine is increasingly recognized as a neuromodulatory factor that extends beyond its classical role as an inflammatory mediator and can influence synaptic function and plasticity (Brietzke and Kapczinski 2008; Takahashi et al. 2021; Kemp et al. 2022). Moreover, accumulating evidence indicates that inflammation can enhance excitability within the amygdala, thereby sensitizing fear‐related neural circuits and ultimately amplifying vigilance and anxiety‐related responses (Takahashi et al. 2021; Hassamal 2023). Taken together, these observations suggest that the anxiety‐like phenotype observed in OTS may not be solely attributable to a psychological response to performance decrements, but may instead reflect, in part, underlying neuroinflammatory processes and maladaptive synaptic changes.

An intriguing observation in the present study is that TJ proteins appear to be differentially regulated according to exercise intensity. Specifically, ZO‐1 emerged as a particularly susceptible component under OT stress, exhibiting a significant reduction in the OT group. ZO‐1 is a core scaffolding protein of the BBB that functions as an anchoring molecule, linking transmembrane proteins such as claudins and occludin to the actin cytoskeleton (Kadry et al. 2020). Consequently, the loss of ZO‐1 might indicate a potential destabilization of the entire junctional complex, which could lead to increased paracellular permeability (Tornavaca et al. 2015; Lochhead et al. 2020). This structural compromise of the BBB may facilitate the infiltration of peripheral cytokines and immune cells into the brain parenchyma, thereby perpetuating central inflammation (Takata et al. 2021; Galea 2021). In this inflammatory context, TNF‐α has been shown to activate the NF‐κB pathway, thereby inducing the expression of matrix metalloproteinase‐9 (MMP‐9), which can promote proteolytic degradation of ZO‐1 (Rajashekhar et al. 2014; Zhao et al. 2022). Furthermore, TNF‐α may trigger phosphorylation of ZO‐1 via the PKCζ pathway, facilitating its dissociation from the plasma membrane (Aveleira et al. 2010; Paul et al. 2015; Lochhead et al. 2020). The parallel elevation of TNF‐α and reduction of ZO‐1 suggests a potential association, indicating that such inflammatory degradation mechanisms could potentially drive BBB impairment in OTS (Varatharaj and Galea 2017; Galea 2021). These results align with reports that systemic administration of TNF‐α can impair BBB integrity (TSAO et al. 2001; Versele et al. 2022; Gao and Bayraktutan 2023), suggesting that the elevated peripheral inflammation observed in our OTS model may serve as a potential driver for the observed ZO‐1 degradation. Notably, among the TJ proteins examined in the present study, occludin protein levels did not differ significantly between groups, whereas ZO‐1 was markedly reduced under OT conditions. Of note, moderate exercise was also associated with a modest reduction in ZO‐1 expression relative to sedentary controls, suggesting that tight junction proteins may undergo dynamic, intensity‐dependent remodeling even within adaptive physiological ranges. This indicates that TJ proteins do not necessarily exhibit parallel or uniform changes and suggests that ZO‐1 may be particularly vulnerable to OT‐induced stress. Therefore, the present results should be interpreted as selective regulation of individual tight junction‐associated proteins rather than evidence of a generalized breakdown of the BBB. This observation is consistent with previous reports showing that the expression levels or immunostaining patterns of individual TJ proteins are often differentially regulated rather than uniformly altered under pathological conditions (Errede et al. 2012; Northrop and Yamamoto 2012; Gao and Bayraktutan 2023). One possible explanation is that, during early stages of barrier disruption, TJ function is regulated primarily through changes in phosphorylation status rather than reductions in total protein abundance (Varatharaj and Galea 2017; Lochhead et al. 2020; Gao and Bayraktutan 2023). Such non‐destructive regulatory changes may be difficult to detect when assessing total protein levels (Varatharaj and Galea 2017). Together, these findings suggest that BBB impairment induced by OT may be characterized by selective vulnerability of ZO‐1, rather than a uniform disruption of the tight junction complex.

In contrast, the ME group exhibited a significant increase in claudin‐5 protein levels. Given that claudin‐5 is a key determinant of BBB size selectivity (Kadry et al. 2020), this finding suggests that moderate‐intensity exercise may enhance BBB integrity (Małkiewicz et al. 2019; Jung 2022). This interpretation is consistent with the concept of hormesis, whereby low levels of physiological stress elicit protective adaptive responses (Calabrese and Mattson 2017). In line with this concept, regular physical exercise has been shown to enhance antioxidative capacity, attenuate oxidative stress, and exert anti‐inflammatory effects, thereby contributing to the maintenance of blood–brain barrier integrity (Małkiewicz et al. 2019). In the OT group, the lack of a beneficial adaptive response suggests that excessive stress intensity may exceed the threshold for positive adaptation, thereby favoring pathological rather than protective pathways. This highlights the critical importance of exercise intensity regulation for maintaining brain health (Armstrong and VanHeest 2002). The present study provides critical insights into the neurobiological impact of excessive training. The association between OT, hippocampal tight junction protein alterations, and neuroinflammation provides a possible framework for understanding mood disturbances in populations exposed to chronic physical stress, such as competitive athletes and military personnel. These findings suggest that anxiety in OTS is not merely a psychological reaction to performance decrements, but may be rooted in neuroinflammatory processes. Therefore, monitoring training intensity is crucial for both physical performance and preserving the mental well‐being of those under chronic stress. However, the present study does not directly demonstrate this mechanistic sequence. Therefore, the proposed pathway should be considered a possible biological framework rather than a demonstrated causal mechanism. Future studies using direct BBB permeability assays, broader neuroinflammatory profiling, and causal interventions will be required to establish this pathway.

### 4.1 Limitations

The present study was conducted exclusively in male mice and therefore did not account for sex dimorphism in anxiety‐ and stress‐related responses. Moreover, training‐related injuries reduced the final OT sample size from 10 to 4 mice. This relatively small sample size may have reduced the statistical power of the analyses and limited the robustness and generalizability of the present findings. In addition, no a priori power analysis was performed before the study. Accordingly, these findings should be interpreted with caution and warrant confirmation in larger cohorts. Additionally, the current analyses were limited to total protein abundance of selected tight junction proteins and did not directly assess BBB permeability or functional integrity. Therefore, the observed changes in ZO‐1 and claudin‐5 should be interpreted as BBB‐related molecular alterations rather than direct evidence of BBB dysfunction. Future studies should include phosphorylation‐specific analyses and direct permeability assessments, such as Evans blue or sodium fluorescein extravasation assays. Neuroinflammation was also evaluated using TNF‐α alone. Future studies should incorporate additional inflammatory cytokines, such as IL‐1β and IL‐6, as well as NF‐κB signaling and microglial activation markers, to provide a more comprehensive assessment of neuroinflammatory responses.

## 5 Conclusion

In conclusion, the present study highlights that exercise intensity serves as a critical determinant in modulating the balance between neuroprotection and neuroinflammatory maladaptation. Our findings suggest that while moderate exercise was associated with increased claudin‐5 expression, which may reflect an adaptive BBB‐related molecular response, excessive training stress could induce a neuroinflammatory milieu and structural destabilization of the barrier.

These observations imply that the anxiety‐like symptoms associated with OTS are not merely psychological phenomena, but may reflect a physiological shift toward neuroinflammation and BBB‐related molecular alterations. Ultimately, this research underscores that precise intensity regulation in exercise programming is vital not only for optimizing physical performance but also for preserving mental well‐being.

## Author Contributions

**Eunseon Hwang**: conceptualization, methodology, data curation, investigation, validation, formal analysis, visualization, project administration, writing – original draft. **Minyoung Shin**: investigation, validation, data curation. **Taeeon Park**: investigation. **Jeremy J. Kim**: investigation. **Dongheon Yi**: conceptualization, methodology. **Jeayeon Park**: investigation, writing – review and editing. **Hyo Youl Moon**: conceptualization, methodology, supervision, funding acquisition, writing – review and editing.

## Funding

This work was supported by the National Research Foundation of Korea (NRF) grant funded by the Korea government (MSIT) (No. RS‐2022‐NR070859, RS‐2025‐16066994 and NRF‐2026S1A5A2A01005211).

## Ethics Statement

All animal experiments were approved by the Institutional Animal Care and Use Committee (IACUC) of Seoul National University (SNU‐240510‐4‐2) and were conducted in accordance with the relevant guidelines and regulations.

## Conflicts of Interest

The authors declare no conflicts of interest.

## Supporting information




**Supplementary Material**: brb371709‐sup‐0001‐SuppMat.docx

## Data Availability

The datasets used and analyzed during the current study are available from the corresponding author on reasonable request.
