# Supplementary material for: The Impact of Overtraining on Anxiety‐Like Behavior and Tight Junction Proteins of the Blood–Brain Barrier in Mice
Source: Brain Behav. 2026 Aug 30;16(9):e71709. doi: 10.1002/brb3.71709 (PMC13527226; doi:10.1002/brb3.71709)
Supplement: Supplementary file 1 [file BRB3-16-e71709-s001.docx]

**Supplementary Table S1. Summary of statistical analyses and effect sizes.**

| **Outcome** | **Statistical test** | **Effect** | **F (df1, df2)** | **P value** | **Effect size** |
| --- | --- | --- | --- | --- | --- |
| Body weight | Two-way RM ANOVA | Time | F(1.260, 13.86)=16.81 | 0.0006 | partial η² = 0.605 |
|  |  | Group | F(2,11)=4.709 | 0.0333 | partial η² = 0.461 |
|  |  | Time × Group | F(2.520,13.86)=0.925 | 0.4399 | partial η² = 0.144 |
| Incremental load test | Two-way RM ANOVA | Time | F(1,11)=0.003 | 0.9542 | partial η² = 0.0003 |
|  |  | Group | F(2,11)=5.405 | 0.0232 | partial η² = 0.496 |
|  |  | Time × Group | F(2,11)=19.92 | 0.0002 | partial η² = 0.784 |
| Grip strength | One-way ANOVA | Group | F(2,11)=2.718 | 0.1098 | η² = 0.331 |
| TA muscle weight | One-way ANOVA | Group | F(2,11)=6.026 | 0.0171 | η² = 0.523 |
| Quadriceps TNF-α | One-way ANOVA | Group | F(2,11)=5.759 | 0.019 | η² = 0.512 |
| OFT: Number of center entries | One-way ANOVA | Group | F(2,11)=6.739 | 0.0123 | η² = 0.551 |
| OFT: Distance in center zone | One-way ANOVA | Group | **F(2,11) = 12.92** | **0.0013** | **η² = 0.701** |
| OFT: Time in center zone | **One-way ANOVA** | **Group** | **F(2,11) = 5.534** | **0.0217** | **η² = 0.502** |
| EPM: Enteries to open arm (%) | **One-way ANOVA** | **Group** | **F(2,11) = 6.116** | **0.0160** | **η² = 0.527** |
| EPM: Distance in open arm | **One-way ANOVA** | **Group** | **F(2,11) = 2.549** | **0.1231** | **η² = 0.317** |
| EPM: Time in the open arm | **One-way ANOVA** | **Group** | **F(2,11) = 1.651** | **0.2360** | **η² = 0.231** |
| Nest-building test | One-way ANOVA | Group | F(2,11)=0.175 | 0.842 | η² = 0.031 |
| ZO-1 | **One-way ANOVA** | **Group** | **F(2,11) = 12.77** | **0.0010** | **η² = 0.699** |
| Occludin | **One-way ANOVA** | **Group** | **F(2,11) = 1.392** | **0.289** | **η² = 0.202** |
| Claudin-5 | **One-way ANOVA** | **Group** | **F(2,11) = 4.890** | **0.030** | **η² = 0.471** |
| Hippocampal TNF-α | **One-way ANOVA** | **Group** | **F(2,11) = 5.759** | **0.0190** | **η² = 0.512** |
